# Distribution of antiretroviral therapy through private pharmacies and postal courier services during COVID‐19 in Botswana: acceptability and reach of two out‐of‐facility individual differentiated service delivery models

**DOI:** 10.1002/jia2.25814

**Published:** 2021-10-28

**Authors:** Mulamuli Mpofu, Tackler Moyo, Masego Gilbert, Wame Dikobe, Lirica Nishimoto, Gorata Katiko, James Batuka, Hind Satti, Maria Qambayot, Hally Mahler, Lesego Kitso, Hannah Marqusee, Moses Bateganya

**Affiliations:** ^1^ FHI 360 Gaborone Botswana; ^2^ FHI 360 Durham NC USA; ^3^ FHI 360 Nairobi Kenya; ^4^ FHI 360 Washington DC USA; ^5^ USAID Gaborone Botswana; ^6^ USAID Washington DC USA

**Keywords:** ARV, Botswana, courier services, COVID‐19, differentiated care, home delivery

## Abstract

**Introduction:**

The advent of COVID‐19 has put pressure on health systems as they implement measures to reduce the risk of transmission to people living with HIV (PLHIV) and healthcare workers. For two out‐of‐facility individual differentiated service delivery (DSD) models, we assessed acceptability of antiretroviral therapy (ART) distribution through private pharmacies and reach of home delivery of ART through courier services during the COVID‐19 pandemic in Botswana.

**Methods:**

From 24 July to 24 August 2020, we conducted exit interviews with PLHIV receiving ART from 10 high‐volume public facilities in Gaborone, and mapped and conducted an online survey with private pharmacies to assess willingness and capacity to dispense ART to PLHIV enrolled in the Botswana national ART program. We piloted ART home delivery from September 2020 to January 2021 in Gaborone and Kweneng East districts for PLHIV accessing ART at two Tebelopele Wellness Clinics. We used cascade analysis to measure the enrolment and eventual reach (percentage of those reached amongst those who are eligible) of ART home delivery.

**Results:**

Sixty‐one PLHIV and 42 private pharmacies participated. Of the PLHIV interviewed, 37 (61%) indicated willingness to access ART from private pharmacies and pay BWP50 (∼US$4) per refill for a maximum of two refills per year. All private pharmacies surveyed were willing to provide ART, and 26 (62%) would charge a dispensing fee (range = BWP50–100; ∼US$4–8) per refill. All pharmacies operated 12 h/day, 6 days/week and on public holidays. In the home delivery pilot, 650 PLHIV were due for refills, 69.5% (*n* = 452) of whom were eligible for home delivery. Of these, 361 were successfully offered home delivery and 303 enrolled (enrolment = 83.9%: female = 87.2%, male = 77.8%, *p* = 0.013). A total of 276 deliveries were made, a reach of 61%.

**Conclusions:**

Providing ART through private pharmacies and home delivery was acceptable in Botswana during COVID‐19. Surveyed pharmacies were willing and able to dispense ART to PLHIV attending public sector facilities for free or for a nominal fee. Additionally, using courier services for ART home delivery is a novel and viable model in countries with a reliable courier service like Botswana and should be scaled up, particularly in urban areas.

## 1 INTRODUCTION

People living with HIV (PLHIV) on antiretroviral therapy (ART) are required to visit healthcare facilities regularly for consultations or medication refills, which has become challenging during the COVID‐19 pandemic. Governments have issued stay‐at‐home orders, curfews and lockdowns, making it difficult to access health services [1, 2]. In Zimbabwe, about 19% of PLHIV who attempted to get their ART refills were not successful during the lockdowns [3]. Similarly, 48% of PLHIV in China did not know how to access their HIV treatment during the COVID‐19 lockdowns [4].

Visiting health facilities during COVID‐19 is high risk due to congestion [5]. The U.S. Centers for Disease Control and Prevention has recommended that “HIV facility visits should be limited to those deemed medically essential, to reduce the risk and burden to recipients of care and health care providers” [6]. For PLHIV, implementation of lockdowns and social distancing measures necessitated urgent enrolment into differentiated service delivery (DSD) models as safe alternatives for accessing ART [7, 8]. Out‐of‐facility individual DSD models for ART including through private pharmacies, home delivery (e.g., courier services) and smart lockers offer alternatives in the context of COVID‐19 [9]. These models offer PLHIV convenient options for continuing treatment, decongest clinics, allowing for physical distancing and safeguarding PLHIV and healthcare workers [1, 10]. Before the COVID‐19 pandemic, the private sector models were implemented on a small scale, primarily to ensure treatment continuity, despite associated cost savings for governments and PLHIV [9].

While there is a dearth of studies on systematic dispensing of ART by private pharmacies and through home delivery to public sector PLHIV in low‐ and middle‐income countries, there are some examples of successful implementation. Noncommunicable disease (NCD) medications have been delivered through courier and other models in South Africa [11, 12]. In Nigeria, PLHIV who utilized private pharmacies for refills had higher treatment continuity rates (88% vs. 73%) and higher viral suppression rates (100% vs. 80%) than those at facilities, while participating clinics were decongested by half [13]. During COVID‐19 restrictions, ART was delivered through courier services to homes of PLHIV who were unable to reach treatment centres in Pakistan [3]; while in Ukraine, home delivery of ART and other medicines were successfully delivered through the country's two biggest postal operators [14]. In India, the postal service delivered drugs following government‐imposed movement restrictions during COVID‐19 [15].

Botswana has the third highest HIV prevalence in the world, with one in five adults aged 15–49 living with HIV [16]. The country adopted the World Health Organization's test‐and‐treat strategy in 2016 and expanded treatment eligibility regardless of CD4 count [17]. The resulting increase in the number of PLHIV on treatment stretched the already constrained public health resources. The country recorded its first confirmed COVID‐19 case on 30 March 2020, and by 21 February 2021, 26,524 cumulative cases and 254 deaths had occurred [18]. Shifting of resources to respond to COVID‐19 has exacerbated existing health system challenges. A national lockdown commenced on 2 April 2020 through May 20 [19]. These measures limited access to ART. To address these challenges in Botswana and other countries, the Meeting Targets and Maintaining Epidemic Control (EpiC) project funded by the U.S. President's Emergency Plan for AIDS Relief (PEPFAR) through the United States Agency for International Development (USAID) provided support to identify and assess the acceptability of alternative ART delivery models and to pilot those deemed feasible. We determined the acceptability of ART distribution through private pharmacies and the reach of home ART delivery through courier services.

## 2 METHODS

### 2.1 Study design and setting

In this study, we assessed two implementation science outcomes: (1) acceptability – defined as the perception among implementation stakeholders that a given treatment, service or innovation is agreeable, palatable or satisfactory [20]; and (2) reach – the absolute number, proportion and representativeness of individuals who participate in a given intervention, and reasons why or why not [21]. To determine the acceptability of the private pharmacy model, we interviewed PLHIV receiving ART from 10 high‐volume public facilities in Gaborone. We also mapped and surveyed private pharmacies proximal to those facilities in Gaborone, Kweneng East and South East districts by administering an online questionnaire using Kobo toolbox [22].

We also designed and piloted delivery of ART to PLHIV's homes or alternative locations in Gaborone and Kweneng East districts through the Botswana Postal Services (BPS), the national courier service. BPS already has an ongoing contract with the Central Medical Stores for warehousing and distribution of drugs to health facilities in the country. Survey tools were adapted from tools developed by EpiC for use in nine countries (including Botswana) which are implementing different decentralized ART models [23]. We then assessed the proportion of eligible PLHIV reached with ART delivered at home or alternative location, through BPS as the main outcome of the pilot.

At the time of the assessment and pilot, out‐of‐facility ART distribution was not national policy. However, the assessments and pilot were authorized by the Botswana Ministry of Health and Wellness (MoHW) to inform national policy. The pilot was conducted at two USAID‐ and PEPFAR‐supported Tebelopele Wellness Clinics (TWC) run by a local implementing partner. Table 2 shows how the proposed DSD models differ from the standard of care.

**Table 1:** Top ten high‐volume ART clinics/facilities in Gaborone District‐June 2020

Name of facility	Number of PLHIV on ART
Nkoyaphiri Clinic	4457
Bontleng Clinic	4434
Broadhurst Traditional Area Clinic	3972
Phase 2 Clinic	3885
Tsholofelo Clinic	3450
Tlokweng Main Clinic	3339
Extension 15 Clinic	2487
Lesirane Clinic	2271
Mogoditshane Clinic	2168
Gaborone West	2133

ART, antiretroviral therapy; PLHIV, people living with HIV.

**Table 2:** Building blocks for standard of care, home and pharmacy ART delivery models

Model	When	Where	Who	What
Standard of care	3 monthly	Public ART clinic	Clinician	ART refill Adherence support
	6 monthly			Clinical consultation ART refill Viral load testing
Home ART delivery	3 monthly	Home	Courier services	ART delivery^a^
	6 monthly	Health facility	Clinician	Clinical consultation ART refill Adherence support^b^ Viral load testing
Proposed pharmacy model	3 monthly	Private pharmacy	Private pharmacist	ART refill Adherence support
	6 monthly	Health facility	Clinician	Clinical consultation ART refill Adherence support^b^ Viral load testing

^a^ ∼50 pula per refill for each client was paid by EpiC for the pilot and later expected by the government or clients (if able and willing).

^b^ Virtual support by clinic 3–6 monthly or as needed.

ART, antiretroviral therapy.

### 2.2 Acceptability of ART distribution through private pharmacies

Acceptability was assessed through exit interviews with PLHIV and through a survey with private pharmacies.

#### 2.2.1 PLHIV exit interviews

From 24 July to 24 August 2020, structured interviews were conducted with PLHIV receiving ART services from the 10 highest‐volume ART facilities in Gaborone. The interviews collected perspectives on ART distribution through private pharmacies, information on travel time to ART sites and to the nearest private pharmacy, waiting time for services, prior use of private pharmacies and interest in receiving refills through them, willingness to pay a dispensing fee and the range of fees they were willing to pay.

The PLHIV were purposively identified and informed about the study on the day they reported for their clinic visit. During recruitment, an EpiC staff member approached them and offered participation. Informed consent was obtained from those who agreed to participate prior to conducting the interview. Participant names were not recorded to ensure confidentiality. Participation was voluntary, and all PLHIV were informed that they could discontinue participation at any time and could decline to respond to any question. After a month, the interviews were stopped to minimize additional risk of COVID‐19 for the interviewers and clients.

#### 2.2.2 Pharmacy survey

The survey was conducted with private pharmacy points of contact. The list of pharmacies, their location and points of contact were obtained from the Pharmacy Society of Botswana (PSB), a professional body of certified pharmacy practitioners. Prior to the selection of the pharmacies, PSB convened its members to sensitize them about the survey.

Using an online questionnaire administered using Kobo toolbox, we assessed their willingness to dispense ART to PLHIV enrolled in Botswana's national ART program; their dispensing fee (refill fee) if at a cost; adequacy of their infrastructure (counselling space, storage space, and security), documentation procedures; operating hours; and staff capacity to support the ART program. Piloting of the private pharmacy DSD model had not commenced at the time of this analysis pending MoHW permission.

### 2.3 Reach of ART home delivery during pilot implementation

With concurrence from MOHW, two community‐based TWCs were purposefully selected in Gaborone (urban) and Kweneng East district (semi‐urban) to pilot ART home delivery through BPS. Tebelopele clinics were established in 2000 as HIV testing centres and in 2019, they started offering integrated HIV services including ART to underserved populations such as men who have sex with men (MSM), female sex workers (FSW), noncitizens and other populations.

Prior to starting home deliveries at TWCs, EpiC and BPS signed a memorandum of understanding for delivery of ART parcels to eligible PLHIV receiving care at the TWCs. BPS was engaged because it was already providing medication warehousing services for the MoHW and delivering parcels in communities where PLHIV reside. As such, ART parcels would not be seen as different from routine packages. A delivery fee of 50 Botswana pula (BWP) (∼US$4), the amount BPS charges for a regular parcel was agreed upon. For this pilot, the delivery fee was paid by EpiC. TWC healthcare workers were trained on how to use the BPS's e‐Waybill, the electronic data capture and parcel tracking system. Antiretroviral (ARV) drugs were packaged in standard BPS‐branded packaging to ensure that they were indistinguishable from other parcels. To maintain confidentiality, BPS staff were also not aware of the specific medication(s) in the parcels.

From September 2020 to January 2021, eligible PLHIV who were established on ART were identified by reviewing their clinic records. PLHIV were considered established on ART if they had been on treatment for more than 6 months, had recent viral load of less than <400 copies/ml, had no current opportunistic infections, as per the Botswana National Treatment Guidelines. Eligible PLHIV were contacted by phone by the TWC nurse and offered the option of receiving their next ARV refill through BPS home delivery. Verbal consent was obtained, and the preferred physical address and time of delivery were confirmed. To prepare for each scheduled delivery, TWC staff completed an electronic form in e‐Waybill and packaged the medications before contacting BPS for pick‐up.

**Figure 1:**
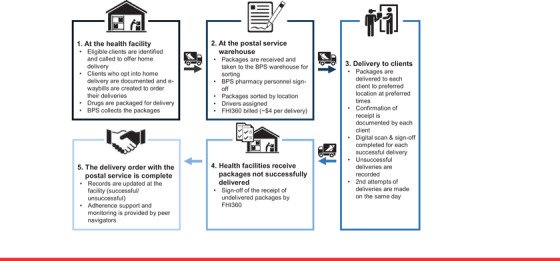
Flowchart of home delivery of ART through Botswana Postal Services (BPS)

Figure 1 shows the home delivery process from eligibility assessment to delivery completion and documentation.

Medication parcels were scheduled for delivery a week before the actual refill due date.

The delivery parcel contained a 3‐month supply of ART, an appointment card for the next clinic or ART refill date and a viral load test request form if a test was due before the next clinic appointment. The refill supply would cover the next 3 months until the next scheduled visit to the facility where they would get their next refill after clinical consultation. Botswana was beginning to transition to 6‐monthly dispensing (6‐ MMD) when the first case of COVID‐19 was reported which was then put on hold to better manage ART stocks given the anticipated shortages. We determined reach by collating the total number of packages that were successfully delivered as a proportion of those that were eligible.

### 2.4 Data analysis

We conducted descriptive analysis to compare frequencies. Additionally, we used Chi Square to test the difference in home delivery acceptability and reach by sex, population group and citizenship. SPSS was used for analysis (IBM Corp. Released 2017. IBM SPSS Statistics for Windows, Version 25.0. Armonk, NY: IBM Corp).

### 2.5 Ethical considerations

The pharmacy assessment received a nonresearch determination from FHI 360 Office of International Research and Ethics. Home delivery processes were nested within the TWCs’ routine activities and aggregate data with no patient identifiers were collected. Confidentiality of medications and other personally identifiable information was further ensured by packing and sealing the medication at the TWCs, and using the same packaging used for all other parcels sent through BPS before it was retrieved by BPS drivers. The contents were not identifiable or known to BPS staff. Additionally, all BPS staff and drivers signed nondisclosure and confidentiality forms.

## 3 RESULTS

### 3.1 PLHIV exit interviews

A total of 61 PLHIV on ART were interviewed from 10 high‐volume facilities in Gaborone. Of these, 57.4% (*n* = 35) were female, 52.4% (*n* = 32) were 40 years and older, and 60.7% (*n* = 37) had been on treatment for more than four years (Table 3).

**Table 3:** Demographic and clinical variable of the ART PLHIV who were interviewed

	Sex					
	Female		Male		Total	
	*n* = 35	%	*n* = 26	%	*N* = 61	%
Age group (years)						
<20	0	0	0	0	0	0
20–29	5	45.5	6	54.5	11	18.0
30–39	110	66.7	6	33.3	18	29.5
40+	190	62.5	12	37.5	32	52.5
Number of years on ART						
0–4	14	40	10	34.6	24	39.3
5–9	13	34.4	10	34.5	23	37.7
10+	8	22.9	6	19.2	14	22.9

ART, antiretroviral therapy; PLHIV, people living with HIV.

Twenty‐six (43%) reported previous private pharmacy use (Table 4).

**Table 4:** PLHIV willingness to use private pharmacies for ARV pick‐up

Measures	Frequency*n* = 61	%
Number of PLHIV who had used private pharmacies previously	26	42.6
PLHIV willing to use private pharmacies	37	60.7
PLHIV willing to use private pharmacies and pay a dispensing fee	27	44.3
Median dispensing fee PLHIV were willing to pay^a^	BWP50 (∼US$4) Range = BWP50–100	

^b^Amongst those willing to pay.

ARV, antiretroviral; PLHIV, people living with HIV.

Of the PLHIV interviewed, 37 (60.67%) indicated willingness to access ART from private pharmacies; this number dropped to 27 (44.2%) if they would be expected to pay a dispensing fee. Amongst those willing to pay, 40% were willing to pay BWP50 (∼US$4) per refill or a maximum of BWP100 per year.

Thirty‐three (54.1%) PLHIV and 52 (85.2%) indicated that they lived within a 30‐minute walking distance to the nearest public ART facility and private pharmacy, respectively. Most PLHIV (*n* = 39; 63.9%) indicated that the waiting time for HIV services at the ART facility was less than 1 h.

### 3.2 Pharmacy survey

Forty‐two private pharmacies in Gaborone, Kweneng East and South East districts participated in the survey (Table 5).

**Table 5:** Results of pharmacy survey and characteristics of participating pharmacies

	Frequency	
	*n* = 42	%
Number of participating pharmacies by district		
Gaborone	33	78.6
Kweneng East	4	9.5
South East	5	11.9
Willingness to support ART distribution		
Number of private pharmacies willing to dispense ARVs on behalf of public facilities	42	100
Number of pharmacies who would charge a dispensing fee	26	61.9
Median dispensing fee	BWP60 (∼US$4)	
Pharmacy capacity		
Pharmacies with qualified pharmacists	42	100
Pharmacies with waiting areas	42	100
Pharmacies with counselling rooms	42	100
Pharmacies already providing ART to private PLHIV	42	100
Pharmacies with latest ART guidelines	26	61.9
Pharmacies with adequate storage capacity	42	100
Median number of pharmacists per pharmacy	1 (Range = 1–5)	
Days of operation		
Weekdays (Monday–Friday)	42	100
Saturdays	42	100
Sundays and public holidays	33	78.5

ART, antiretrovial therapy; ARVs, antiretrovirals; PLHIV, people living with HIV.

All private pharmacies were willing to provide ART on behalf of public facilities, although 26 (62%) indicated that they would require a dispensing fee of BWP60 [range: BWP50–100; (∼US$ 4–8)] per refill, either paid directly by the PLHIV or by the MoHW. All 42 pharmacies were already dispensing ART to private clients. They reported having adequate space in their waiting area and a designated private area for counselling. They also operated at least 12 h per day on weekdays and were open on Saturdays. However, only 33 (78.5%) operated on Sundays and public holidays for limited hours.

### 3.3 ART home delivery pilot

**Figure 2:**
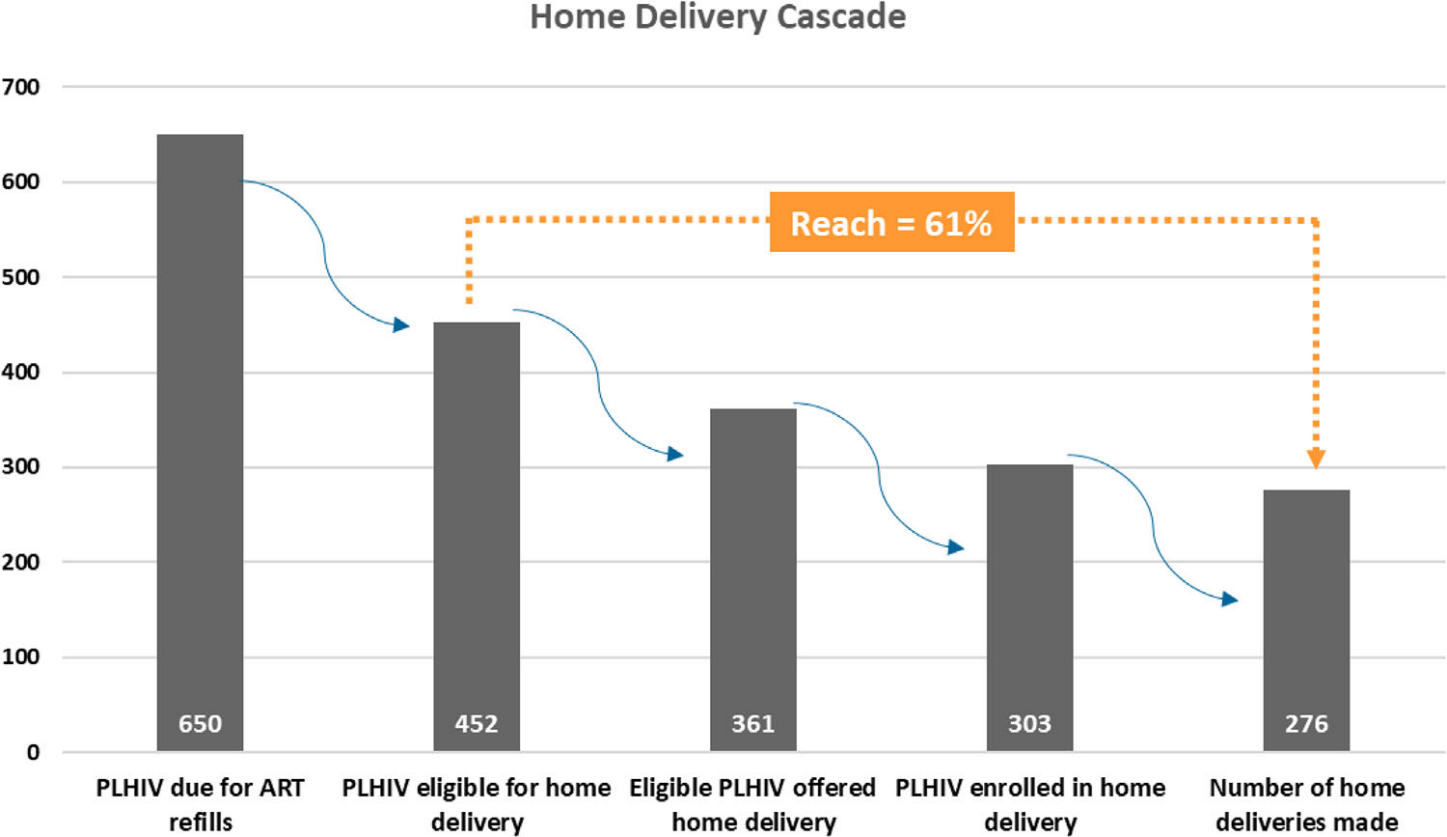
Cascade of home delivery of ART through BPS in Botswana, 22 September 2020–12 February 2021

A total of 650 PLHIV were identified as due for refills for the period 22 September 2020–12 February 2021 in the two pilot clinics (Figure 2). Among those due for refills, 69.6% (452 out of 650) were found to be eligible for home delivery, 79.8% (361 out of 452) were successfully offered home delivery through BPS. The remaining 20.2% could not be contacted by phone.

Out of those who were eligible, the percentage reached with home deliveries was 61% although 83.9% (303 out of 361) initially enrolled. Of those offered home delivery, 13.6% (49 out of 361) were not interested at all, while an additional 13 (3.6%) were undecided or needed to consult with their partners. Reasons for declining home delivery included preference for the facility pickup (30%), was mobile with no stable delivery address (19%) and workplace constraints (15%). Twenty‐seven (8.9%) parcels were returned to the facility because recipients were not at home or had decided to pick up their ART at the health facility before the delivery was made.

The enrolment for home delivery was higher amongst females (AR = 87.2%) than males (AR = 77.8%, *p* = 0.01) (Table 6). Enrolment was statistically equivalent between the general population and key population (KP) (specifically, MSM and FSW) (*p* = 0.447) and by citizenship (*p* = 0.52).

**Table 6:** Enrolment for home delivery of ART by sex, population group and citizenship

	Home delivery enrolment			
	Enrolled	Refused	Enrolment	*p* value
Sex				0.013
Females	184	27	87.2%	
Males	119	34	77.8%	
Total	303	61	100%	
Population group				0.447
General population	135	26	83.9%	
Key population^a^	168	35	82.8%	
Citizenship				0.517
Botswana citizens	142	29	83.0%	
Noncitizens	161	32	83.4%	

^a^MSM, FSW.

## 4 DISCUSSION

We found high interest and acceptability of the pharmacy and home delivery models in Botswana, demonstrating that these models could ensure treatment continuation in the context of COVID‐19. Botswana has made significant progress in its HIV response [24] with high treatment continuation (95%) and viral suppression rates (97%) [16]; an estimated 310,000 PLHIV are currently on ART [16], the majority through a few high‐volume facilities. The burden on these facilities can be reduced by adding ART home delivery and private pharmacy distribution to current DSD models. The advent of COVID‐19 coupled with the growing number of PLHIV established on ART in Botswana calls for the implementation of these innovative models that leverage the private sector.

Our assessment revealed several opportunities. First, private pharmacies in Botswana are already distributing NCD medications and are dispensing ART for private clients who pay out of pocket. They are willing and have the infrastructure to serve PLHIV enrolled in the national program. The high willingness to access ART refills through private pharmacies is encouraging considering that the majority of Batswana living with HIV access ART services primarily through public facilities.

Second, the 50 BPS post offices already store and distributes medications (including ARVs) to regional distribution centres and hospitals. This thriving parastatal could be leveraged to support home delivery of ART. Overall, 83.9% of PLHIV who were offered home delivery of ART accepted, highlighting the potential for a courier DSD model. The home delivery model has long been a preferred method in other countries [25]. Expanding this model in Botswana would address stigma, since many people already receive their NCD medication through this approach. During the COVID‐19 pandemic, the home delivery model can decongest public health facilities and, therefore, minimize the risk of COVID‐19 to both PLHIV and healthcare providers. Furthermore, the decentralization of ART improves continuity in care [26]. Decentralization through the private sector has the potential of cost savings for governments, donors and, more importantly, PLHIV, as evidenced through modelling data [7].

DSD empowers PLHIV to find a model of care conducive to their lifestyle while decongesting the healthcare system [27]. The assessment provides data which can inform the design of more PLHIV‐centred services. About 85% of PLHIV surveyed lived within 30‐minute walking distance to a private pharmacy, thus using them as pick up points for out‐of‐facility individual DSD models could put services within convenient reach. All private pharmacies assessed had resident pharmacists, an attribute that will assure high‐quality ART services. In addition, private pharmacies and home delivery could address the issue of waiting time for HIV services at heath facilities, which can be substantial at high‐volume facilities [28]. The home delivery cuts down on travel costs and time spent at health facilities. Coupled with multi‐month dispensing, these models would reduce the number of clinic visits and associated costs [25].

User fees are a major barrier to accessing services in both the public and private sectors for the majority of PLHIV [29, 30]. Our assessment found that less than half of the PLHIV were willing to obtain their ART refills from private pharmacies if they had to pay a dispensing fee. Importantly, the ART dispensing fee which was proposed by the private pharmacies was at par with the median fee proposed by PLHIV, indicating the viability of this option. Including user fees in the design of private sector programs increases their sustainability, reduces the need for significant donor or government subsidies but can result in lower uptake of services. However, the cost to PLHIV could be lowered by creating a business case with pharmacies and appealing to their corporate social responsibility. We also found through the pilot that the cost of home delivery was about the same as the dispensing fee proposed by pharmacies (∼US$4 vs. US$5, respectively), making these two models roughly equivalent in cost. Educating PLHIV on each option's relative advantages and evaluating the models side by side would provide the government of Botswana with the relevant information for large‐scale rollout.

The acceptability of the home delivery model was significantly higher amongst females, consistent with acceptability of other HIV services such as HIV testing, whose uptake tends to be lower amongst men [31, 32]. Our finding that there was no difference in acceptability rates by citizenship is positive, as the Botswana government aims to ensure equity in delivery of HIV services. The home delivery success rate in our pilot was high, at 91.1%. We ascribe this success to staff adherence to procedures, including contacting PLHIV prior to delivery and delivering the parcels at a time and place of their choosing.

Home delivery can also address stigma and discrimination faced by key population (KP). Though we did not find any significant difference in the acceptability of home delivery between the general population and KP (83.9% vs. 82.8%), it is well established that KP often experience stigma and discrimination in healthcare settings, resulting in decreased access to services [33, 34]. DSD models have been used to address this gap and enhance access [35]. However, the pilot was not designed to address this issue.

This assessment had some limitations. First, the use of private pharmacies for distribution of ART had not begun at the time of the study, and we were not able to compare the performance of this model with that of home delivery. Second, our sample size was small, in part due to limitations related to COVID‐19. The pharmacies assessed were also located in urban areas. These models might apply differently to rural and urban settings, where acceptability may also vary. Last, the findings may have limited generalizability because of smaller sample sizes and geographical coverage.

During this analysis, discussions with the MoHW in Botswana were ongoing to expand the home delivery model beyond the TWCs to public health facilities, through private pharmacies and smart lockers. The MoHW is cognizant of its responsibility to pay for HIV services and is carefully reviewing lessons from the pilot as well as the cost implementations before making policy decisions or allowing further geographical expansion. EpiC is continuing to work alongside the MoHW to ensure seamless integration and national roll‐out of these out‐of‐facility DSD models. MoHW approval could pave the way to maximize the potential of the more than 350 private pharmacies in the country, the 50 BPS post offices and their distribution networks for distribution of ART and other medications.

## 5 CONCLUSIONS

Provision of ART through private pharmacies is acceptable to both PLHIV and the private pharmacy providers in Botswana. While the pharmacies would prefer to charge a fee, the cost is within the range PLHIV are also willing to pay making it feasible to implement this private sector model beyond NCD drugs which are distributed through private pharmacies on behalf of government. An alternative and novel model of using courier services for ART is viable in countries with a reliable courier service like Botswana and should be scaled up, particularly in urban areas. Scaleup of these models would decongest public health facilities, safeguard staff and PLHIV against COVID‐ 19, and free up space, financial and human resources to address the COVID‐19 pandemic.

## COMPETING INTERESTS

The authors declare no competing interests.

## AUTHORS’ CONTRIBUTIONS

MM, TM, MG, WD and MB led the conceptualization of the paper and the writing of the methods section. GK, TM and MQ led the extraction, validation and cleaning of the program data and contributed towards data analysis and the writing of the methods and results sections. MM led the data analysis and writing of the introduction, results and discussion. MQ, JB, HS and MB wrote part of the introduction, the methods and the results, and LN contributed to writing the methods section. LK, HM and HMQ reviewed all sections of the manuscript. All authors read and approved the final manuscript.

## FUNDING

The EpiC Botswana project is implemented with funding from PEPFAR through USAID (agreement number 7200AA19CA00002) and in collaboration with the Botswana Ministry of Health and Wellness.
